# Carbonic anhydrase VIII as a regulator of intracellular calcium signaling: mechanistic insights and implications in human disease

**DOI:** 10.1007/s11033-026-12124-y

**Published:** 2026-06-10

**Authors:** Ashok Aspatwar

**Affiliations:** Faculty of Medicine and Health Technology, Tampere University, Tampere, 33014 Finland

**Keywords:** Carbonic anhydrase VIII, Pseudoenzyme, Calcium signaling, IP3 receptor (IP3R1), Neuronal signaling, Cerebellar ataxia, Protein–protein interactions, Neurodevelopment, Purkinje cells

## Abstract

Carbonic anhydrases are classically defined as zinc-dependent enzymes that catalyze the reversible hydration of carbon dioxide and play essential roles in acid–base homeostasis. However, carbonic anhydrase VIII (CA VIII) represents a catalytically inactive member of this family due to the absence of one of the three histidine residues required for enzymatic activityStructural analyses further indicate that, despite this loss of catalytic function, CA VIII retains the conserved carbonic anhydrase fold, suggesting functional repurposing rather than structural degeneration. Despite this catalytic silencing, CA VIII is evolutionarily conserved and highly expressed in neuronal tissues, indicating important biological functions beyond enzymatic catalysis. Converging structural, biochemical, and genetic evidence supports the view that CA VIII functions as a pseudoenzyme that regulates intracellular signaling pathways. Mechanistically, CA VIII interacts with the inositol 1,4,5-trisphosphate receptor type 1 (IP3R1), modulating intracellular calcium release and thereby influencing neuronal excitability, synaptic function, and cerebellar development. Disruption of CA VIII has been consistently associated with altered calcium homeostasis and impaired neuronal signaling. Importantly, human genetic studies provide direct evidence for the physiological significance of CA VIII. Mutations identified in affected families, including reports from Iraqi and Saudi Arabian populations, are associated with cerebellar ataxia and neurodevelopmental abnormalities, linking CA VIII dysfunction to human disease. These findings establish CA VIII as a critical regulator of neuronal calcium signaling with clear clinical relevance. In this review, we integrate structural, molecular, and genetic evidence to position CA VIII as a functionally repurposed pseudoenzyme that operates as a signaling regulator rather than a catalytic enzyme. We further discuss its emerging roles in neuronal physiology and disease, and highlight broader implications for pseudoenzyme biology and the evolution of protein function.

## Introduction

Carbonic anhydrases (CAs) constitute a large family of zinc-containing metalloenzymes that catalyze the reversible hydration of carbon dioxide and play essential roles in diverse physiological processes, including pH regulation, ion transport, and cellular metabolism [1]. In contrast to catalytically active isoforms, carbonic anhydrase-related proteins (CARPs)—including CA VIII, CA X, and CA XI—lack enzymatic activity due to the absence of key zinc-coordinating residues within their active sites, yet remain evolutionarily conserved across species [2–4]. This paradox of structural conservation despite catalytic inactivity has prompted increasing interest in understanding their non-canonical biological functions.

Among these, carbonic anhydrase VIII (CA VIII) has emerged as a particularly intriguing member due to its predominant expression in the central nervous system, especially in cerebellar Purkinje cells, where it plays a critical role in neuronal development and signaling [5–7]. CA VIII is highly expressed in cerebellar Purkinje cells, whereas other carbonic anhydrase-related proteins, including CA X and CA XI, show broader expression patterns in the brain and other tissues during development. Structural studies have demonstrated that CA VIII retains the overall fold characteristic of active carbonic anhydrases, but suggest an evolutionary shift from enzymatic to regulatory function [8–10].

Functionally, CA VIII has been shown to interact with IP3R1 and modulate its activity, a key mediator of intracellular calcium release, thereby implicating it in the modulation of calcium signaling pathways essential for neuronal excitability and synaptic plasticity [11, 12]. Disruption of this interaction has been associated with altered calcium homeostasis and impaired neuronal function, highlighting its significance in maintaining cerebellar integrity [13].

Importantly, human genetic studies have established a direct link between CA8 mutations and neurological disorders, particularly cerebellar ataxia and related syndromes, underscoring its critical role in neurodevelopment and motor coordination [14–17]. Beyond the nervous system, emerging evidence suggests that CA VIII may also contribute to tumor progression and metabolic regulation, indicating broader systemic roles that extend beyond its classical neuronal functions [3, 7, 18, 19].

Together, these findings position CA VIII as a non-catalytic yet functionally significant protein that operates at the interface of structural biology, intracellular signaling, and human disease. In this review, we synthesize current knowledge on the structural basis of its catalytic inactivation, its mechanistic role in calcium signaling, and its emerging implications in human pathophysiology.

## Expression and tissue distribution of CA VIII

Carbonic anhydrase VIII (CA VIII) exhibits a highly characteristic expression pattern in the nervous system, particularly within the cerebellum. Early expression studies demonstrated that CA8 transcripts are strongly enriched in cerebellar Purkinje cells, whereas expression in other regions of the brain is comparatively lower [3, 20]. Immunohistochemical analyses further confirmed prominent localization of CA VIII protein in Purkinje neurons in both mouse and human cerebellum, supporting a specialized role in cerebellar physiology and motor coordination [2, 21]. In addition to the cerebellum, lower levels of expression have also been reported in the cerebrum, brainstem, and several peripheral tissues including lung, liver, stomach, salivary gland, colon, and kidney, indicating that CA VIII may possess broader physiological functions beyond the central nervous system [2, 3].

Developmental studies suggest that CA VIII expression is tightly linked to neuronal maturation and cerebellar development. Expression of Car8 has been detected during embryonic stages in mice and zebrafish, supporting an evolutionarily conserved role in neural development [5, 21]. In zebrafish, CA8 expression was observed throughout early developmental stages and was particularly associated with the nervous system and cerebellar structures [21]. Functional suppression of CA8 in zebrafish resulted in abnormal cerebellar morphology, neuronal cell death, and impaired motor coordination, emphasizing the importance of CA VIII during vertebrate neurodevelopment [21]. Additional studies in mouse models demonstrated that CA VIII deficiency alters synaptic organization and excitatory signaling within Purkinje cells, further linking its expression pattern to cerebellar circuitry and neuronal function [13].

Compared with CA VIII, the related acatalytic isoforms CA X and CA XI display broader distribution patterns within the central nervous system [2, 3]. Real-time quantitative PCR and immunohistochemical analyses demonstrated widespread expression of Car10 and Car11 transcripts in several brain regions, whereas CA VIII expression remains particularly enriched in cerebellar Purkinje cells [2]. This differential expression pattern suggests that the CARP family members may possess partially distinct but complementary roles in neuronal signaling and brain development.

## Structural basis of catalytic inactivation

Carbonic anhydrase VIII (CA VIII) belongs to the α-carbonic anhydrase family and retains the overall three-dimensional fold characteristic of catalytically active isoforms. However, unlike classical carbonic anhydrases, CA VIII lacks enzymatic activity due to critical alterations within its active site that prevent coordination of the catalytic zinc ion [8–10]. In catalytically active α-carbonic anhydrases, zinc coordination is mediated by three conserved histidine residues essential for catalysis. In CA VIII, substitution of one of these critical histidine residues disrupts the canonical zinc-binding geometry, thereby abolishing catalytic activity [7, 10].

**Fig. 1:**
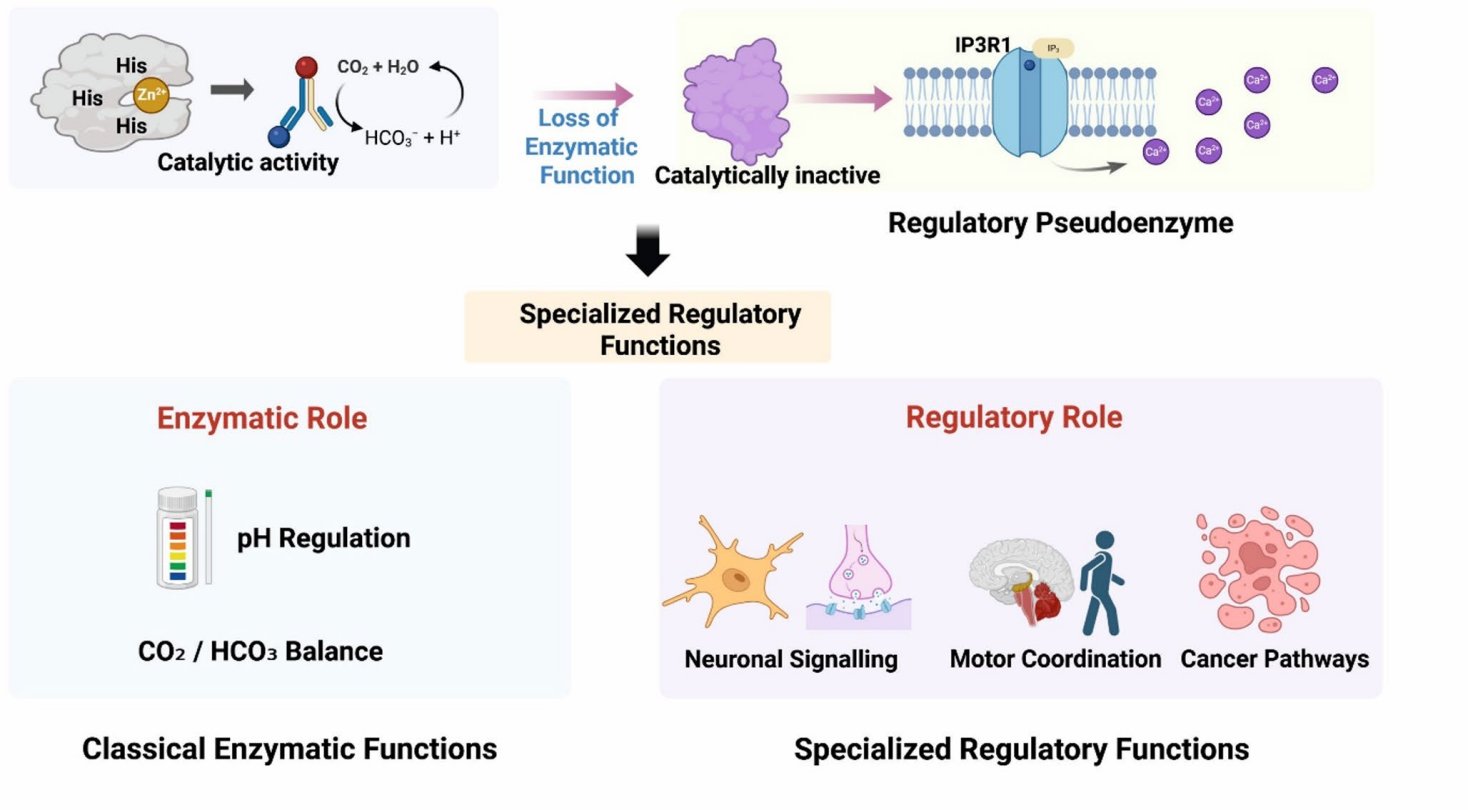
Evolutionary repurposing of carbonic anhydrase VIII from a catalytic enzyme to a regulatory pseudoenzyme. Carbonic anhydrase VIII (CA VIII) has evolved from an ancestral catalytically active α-carbonic anhydrase that mediates the reversible hydration of carbon dioxide (CO₂ + H₂O ⇌ HCO₃⁻ + H⁺) through a zinc-dependent mechanism. In catalytically active enzymes, Zn²⁺ is coordinated by conserved histidine residues within the active site, enabling efficient proton transfer and acid–base regulation. During evolution, substitutions in key Zn²⁺-binding histidine residues led to the loss of catalytic activity in CA VIII. Despite this inactivation, the overall carbonic anhydrase structural scaffold is preserved, allowing CA VIII to retain structural stability and acquire new functional roles. This transition represents a shift from an enzymatic to a non-enzymatic mode of action. In its repurposed state, CA VIII functions as a regulatory pseudoenzyme that interacts with signaling proteins, including the inositol 1,4,5-trisphosphate receptor type 1 (IP3R1), to modulate intracellular Ca²⁺ signaling. This functional shift enables CA VIII to participate in higher-order biological processes such as neuronal signaling, cerebellar motor coordination, and potentially cancer-related pathways. The figure illustrates the evolutionary trajectory of CA VIII, highlighting the transition from catalytic activity to regulatory function, and underscores the concept that loss of enzymatic activity can drive the emergence of novel signaling roles

High-resolution structural analyses have demonstrated that, despite this loss of catalytic function, CA VIII maintains a highly conserved backbone architecture, including the central β-sheet core and surrounding α-helices typical of the carbonic anhydrase fold [10]. This structural preservation suggests that evolutionary pressure has retained the protein’s conformation for functions independent of enzymatic activity (Fig. 1). Indeed, comparative modeling and mutational analyses have shown that the active-site cavity remains intact but is repurposed, potentially serving as a platform for protein–protein interactions rather than substrate binding [8].

Further insight into the structural basis of catalytic silencing has been obtained through studies demonstrating that restoration of zinc-coordinating residues can partially rescue enzymatic activity in carbonic anhydrase-related proteins, including CA VIII [9]. These findings confirm that the loss of catalysis is not due to global misfolding but rather to specific, evolutionarily selected substitutions within the active site. Additionally, analyses of missense variants in CA VIII have revealed that mutations affecting structural stability or local folding can disrupt its regulatory functions, further emphasizing the importance of structural integrity for its biological role [8]. Collectively, these observations support the concept that CA VIII represents a structurally conserved but functionally repurposed protein, in which catalytic inactivation has enabled the emergence of novel regulatory roles (Fig. 1). Rather than acting as an enzyme, CA VIII appears to function as a molecular scaffold, leveraging its preserved architecture to participate in intracellular signaling networks.

## CA VIII as a regulator of intracellular calcium signaling

A defining functional feature of carbonic anhydrase VIII (CA VIII) is its role in the regulation of intracellular calcium signaling, primarily through its interaction with the inositol 1,4,5-trisphosphate receptor type 1 (IP3R1), a major calcium release channel located on the endoplasmic reticulum [11, 12]. Unlike classical carbonic anhydrases that modulate cellular physiology through enzymatic activity, CA VIII exerts its effects via direct protein–protein interaction, positioning it as a non-catalytic regulator of intracellular signaling pathways.

**Fig. 2:**
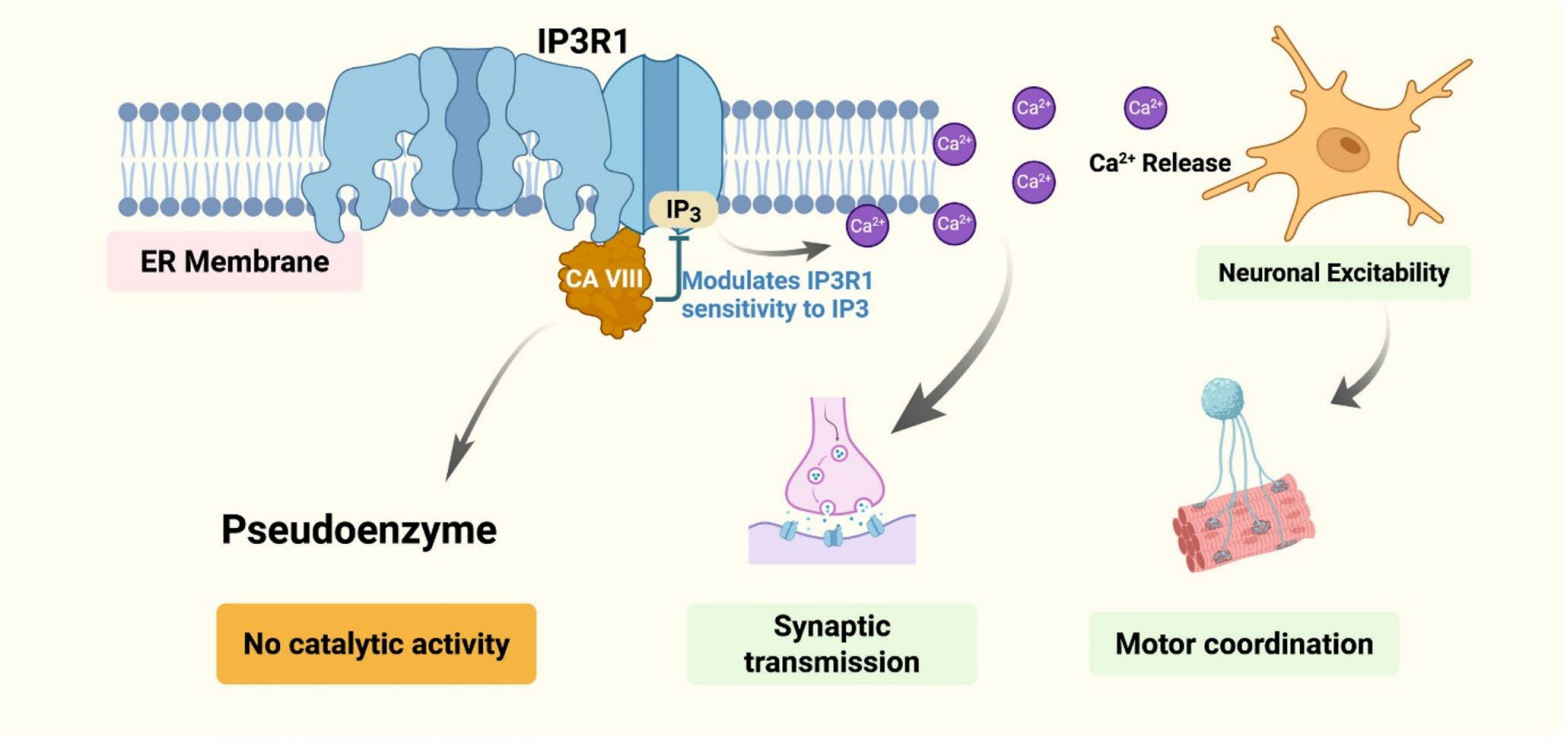
CA VIII as a regulator of IP3R1-mediated calcium signaling. Carbonic anhydrase VIII (CA VIII) functions as a catalytically inactive member of the carbonic anhydrase family and acts as a regulatory pseudoenzyme. Unlike enzymatically active carbonic anhydrases, CA VIII lacks catalytic activity but retains the structural framework required for protein–protein interactions. CA VIII interacts with the inositol 1,4,5-trisphosphate receptor type 1 (IP3R1), a calcium release channel located on the endoplasmic reticulum (ER) membrane. Through this interaction, CA VIII modulates IP3R1 activity, thereby regulating the release of Ca²⁺ from the ER into the cytosol. This controlled calcium signaling is essential for neuronal processes, including neuronal excitability, synaptic transmission, and motor coordination. Disruption of CA VIII function leads to dysregulated intracellular Ca²⁺ signaling, contributing to impaired neuronal communication and motor coordination defects. The figure illustrates the non-enzymatic regulatory role of CA VIII in shaping intracellular calcium dynamics and highlights its importance in neuronal signaling pathways

Biochemical and functional studies have demonstrated that CA VIII directly interacts with the modulatory domain of IP3R1 rather than binding IP3 itself, thereby reducing the sensitivity of IP3R1 to IP3 and influencing calcium release dynamics within neurons [11]. This interaction is particularly critical in cerebellar Purkinje cells, where tightly regulated calcium signaling is essential for neuronal excitability, synaptic integration, and motor coordination. Mechanistically, CA VIII is thought to act as a negative regulator of IP3R1, reducing excessive calcium release and maintaining intracellular calcium homeostasis [12] (Fig. 2).

Disruption of CA VIII function leads to dysregulated calcium signaling, which has been consistently observed in both experimental models and human disease contexts. In CA VIII-deficient waddles (wdl) mice, cerebellar gene expression profiling revealed dysregulation of genes involved in intracellular signaling, synaptic vesicle formation and transport, calcium and zinc ion binding, synaptic integrity, and neuronal development [13]. Notably, altered expression of genes associated with Purkinje cell signaling, GABA receptor subunits, Golgi apparatus function, and synaptic plasticity further supported the role of CA VIII in maintaining cerebellar neuronal homeostasis [13]. Furthermore, detailed analyses of disease-associated mutations affecting the IP3R1 pathway have reinforced the central role of CA VIII in modulating calcium channel activity and maintaining neuronal stability [11].

At the cellular level, studies using neuronal systems have shown that perturbation of CA VIII expression alters calcium-dependent signaling cascades, affecting processes such as neurotransmitter release, synaptic plasticity, and neuronal survival [22, 23]. Consistent with this regulatory role, disruption of CA VIII in zebrafish models leads to altered neuronal development and motor dysfunction, providing in vivo evidence for the functional importance of CA VIII-mediated calcium signaling pathways [21]. These findings collectively support a model in which CA VIII functions as a molecular regulator that fine-tunes intracellular calcium dynamics rather than directly generating biochemical signals. Importantly, this regulatory role provides a mechanistic explanation for the neurological phenotypes associated with CA8 mutations. Given the central importance of calcium signaling in neuronal function, even subtle disruptions in CA VIII-mediated modulation of IP3R1 can lead to profound effects on cerebellar circuitry and motor coordination. The proposed regulatory mechanisms of CA VIII in intracellular calcium signaling are summarized in Fig. 2.

Taken together, CA VIII represents a paradigm shift within the carbonic anhydrase family, functioning not as an enzyme but as a critical modulator of intracellular signaling networks. Its interaction with IP3R1 establishes a direct mechanistic link between structural protein evolution and functional specialization in neuronal systems.

## Evidence from human genetics and disease

The physiological importance of carbonic anhydrase VIII (CA VIII) is most convincingly demonstrated through human genetic studies, which have established a direct link between *CA8* mutations and neurological disorders, particularly cerebellar ataxia and related syndromes [14–17]. These findings provide strong in vivo evidence that CA VIII is essential for normal cerebellar function and motor coordination. Importantly, experimental animal models further support the conserved role of CA VIII in neurodevelopment. In a zebrafish model, morpholino-mediated knockdown of ca8 resulted in pronounced developmental abnormalities, including a curved body axis, pericardial edema, and impaired motor behavior. Histological and ultrastructural analyses revealed significant defects in cerebellar architecture and increased neuronal cell death, closely resembling the ataxic phenotype observed in humans. These findings demonstrate that CA VIII is essential for normal cerebellar development and motor coordination and highlight an evolutionarily conserved role of CA VIII in neuronal function [21, 24, 25].

Initial reports identified homozygous mutations in the *CA8* gene in patients presenting with a syndrome characterized by ataxia, mild intellectual disability, and, in some cases, quadrupedal gait, highlighting a critical role for CA VIII in neurodevelopment [14]. Subsequent studies have expanded the phenotypic spectrum associated with CA8 mutations, revealing variability in disease severity, age of onset, and associated neurological features, including hypotonia, dysarthria, and impaired motor learning [15–17]. These observations suggest that CA VIII function is not only essential but also finely tuned, with even partial loss of function leading to measurable neurological deficits.

Several disease-associated CA8 variants have provided important genotype–phenotype correlations linking specific molecular alterations to neurological dysfunction. For example, the homozygous S100P substitution identified in patients with cerebellar ataxia and mild intellectual disability was shown to markedly reduce CA VIII protein stability, leading to functional deficiency despite preservation of the overall protein framework [14]. Additional truncating and missense variants identified in affected individuals have been associated with variable clinical manifestations, including hypotonia, dysarthria, impaired motor coordination, and quadrupedal gait [15–17]. These findings suggest that distinct mutations affecting CA VIII stability, folding, or interaction with IP3R1 can differentially influence disease severity and neurological phenotype.

At the molecular level, many of the identified mutations affect protein stability, folding, or interaction capacity rather than inducing gross structural disruption, consistent with the notion that CA VIII functions as a regulatory protein rather than an enzyme [8, 16]. This is further supported by studies linking disease-associated variants to altered interaction with IP3R1, reinforcing the central role of CA VIII in calcium signaling pathways that are critical for neuronal function [11].

In addition to inherited mutations, autoimmune mechanisms targeting CA VIII have also been implicated in neurological disease. Autoantibodies against CA VIII have been identified in patients with paraneoplastic cerebellar degeneration, suggesting that disruption of CA VIII function—whether genetic or immune-mediated—can lead to similar pathological outcomes [26–28]. This convergence of genetic and immunological evidence underscores the importance of CA VIII in maintaining cerebellar integrity.

Beyond neurological disorders, emerging evidence suggests that CA VIII may participate in broader cellular processes, although these roles remain incompletely understood. For example, alterations in CA8 expression have been reported in cellular models associated with mitochondrial dysfunction and stress responses, indicating potential links to cellular homeostasis [7]. However, these associations remain indirect, and the strongest and most consistent evidence continues to support a central role for CA VIII in neuronal signaling.

Collectively, human genetic and clinical studies firmly establish CA VIII as a key regulator of neuronal function, with its disruption leading to well-defined disease phenotypes. These findings provide a crucial link between molecular mechanism and clinical manifestation, reinforcing the concept that CA VIII operates as a non-catalytic signaling regulator rather than a conventional enzyme. A comprehensive summary of genetic, experimental, and clinical evidence supporting CA VIII function is presented in Table 1.

**Table 1:** Genetic, experimental, and clinical evidence supporting CA VIII function

Category	System / Model	Key Findings	Functional Insight	References
Human genetics	Iraqi family (consanguineous)	Homozygous *CA8* mutation associated with cerebellar ataxia	Loss of CA VIII disrupts neuronal signaling	[14]
Human genetics	Saudi Arabian family	*CA8* mutation segregates with motor dysfunction and developmental delay	Confirms role in cerebellar function	[15]
Animal model	Waddles mouse (*Car8* mutant)	Motor coordination defects and cerebellar abnormalities	Essential for Purkinje neuron function	[13]
Animal model	Zebrafish	Disruption of *ca8* affects neuronal development and behavior	Conserved role in neurodevelopment	[22]
Cellular studies	Neuronal cells	CA VIII interacts with IP3R1 and modulates IP3R1-mediated Ca²⁺ release	Regulates intracellular calcium signaling	[11, 22, 23]
Molecular mechanism	Structural/biochemical studies	Substitution of Zn²⁺-binding histidine residues abolishes catalytic activity	Structural scaffold retained for regulatory interactions	[8–10]
Clinical evidence	Paraneoplastic cerebellar disease	Autoantibodies against CA VIII detected	Immune-mediated disruption of neuronal signaling	[26–28]
Cancer studies	Multiple cancers	Altered CA VIII expression linked to proliferation and tumor progression	Potential role in tumor-related signaling pathways	[29–33]

## CA VIII in cancer and emerging systemic roles

Beyond its well-established role in neuronal signaling, carbonic anhydrase VIII (CA VIII) has increasingly been implicated in cancer biology and broader cellular processes, suggesting that its regulatory functions extend beyond the nervous system. Although originally characterized as a brain-enriched protein, accumulating evidence indicates that CA VIII expression is dysregulated in multiple tumor types, where it appears to contribute to tumor progression, cell proliferation, and invasiveness [29–33].

Several studies have reported elevated expression of CA VIII in colorectal cancer, lung adenocarcinoma, and osteosarcoma, with functional analyses demonstrating that increased CA VIII levels promote tumor cell growth and survival [29, 31, 32]. In particular, CA VIII has been shown to enhance cell proliferation and support oncogenic signaling pathways, although the precise molecular mechanisms remain incompletely defined. In lung cancer models, CA VIII overexpression has been associated with increased invasive potential, suggesting a role in tumor metastasis [30]. Similarly, studies in renal cell indicate a potential role for CA VIII in modulating signaling pathways associated with tumor progression, tumor cell migration, and proliferation, further supporting its involvement in cancer progression [33].

Mechanistically, these effects are likely mediated through CA VIII’s capacity to modulate intracellular signaling pathways rather than enzymatic activity. Given its established role in calcium signaling, it is plausible that CA VIII contributes to oncogenic processes by influencing calcium-dependent pathways that regulate cell cycle progression, apoptosis, and cytoskeletal dynamics. Additionally, emerging evidence suggests that CA VIII may interact with microRNA-regulated networks and transcriptional pathways, further expanding its role as a signaling modulator in cancer cells.

In parallel, studies have begun to uncover broader systemic functions of CA VIII and related proteins. Notably, carbonic anhydrase-related proteins have been shown to facilitate lactate transport through interaction with monocarboxylate transporters such as MCT1, highlighting a potential role in cellular metabolism and metabolic adaptation [18]. This finding is particularly relevant in the context of cancer, where metabolic reprogramming is a hallmark of disease progression. By influencing metabolite transport and intracellular signaling, CA VIII may act as a bridge between metabolic regulation and signaling networks.

Despite these emerging insights, the role of CA VIII in cancer and systemic physiology remains less well defined compared to its function in the nervous system. Current evidence is largely based on expression analyses and in vitro studies, and further mechanistic investigations are required to establish causal relationships and delineate precise signaling pathways. Nevertheless, the available data support the concept that CA VIII functions as a multifunctional regulatory protein with context-dependent roles across different tissues.

Taken together, these findings extend the functional landscape of CA VIII beyond neuronal biology, positioning it as a potential contributor to cancer progression and metabolic regulation. This broader perspective reinforces the emerging view of CA VIII as a versatile signaling regulator whose impact is not limited to a single physiological system.

## Conclusion

Carbonic anhydrase VIII (CA VIII) represents a compelling example of evolutionary functional divergence within the carbonic anhydrase family, transitioning from a catalytically inactive protein to a critical regulator of intracellular signaling. Despite lacking enzymatic activity, CA VIII retains a highly conserved structural framework that enables it to participate in protein–protein interactions, most notably with the inositol 1,4,5-trisphosphate receptor type 1 (IP3R1), thereby modulating intracellular calcium dynamics [11, 12].

Mechanistic studies have established that CA VIII plays a central role in maintaining calcium homeostasis in neuronal cells, particularly within cerebellar Purkinje neurons, where precise regulation of calcium signaling is essential for normal motor coordination and synaptic function. Disruption of this regulatory axis—whether through genetic mutations or autoimmune targeting—leads to well-defined neurological phenotypes, including cerebellar ataxia, underscoring the physiological importance of CA VIII [14–17, 26–28].

Beyond the nervous system, emerging evidence suggests that CA VIII may contribute to tumor progression and metabolic regulation [29–33]. Although these roles remain less well characterized, they point toward a broader functional repertoire in which CA VIII integrates signaling and metabolic pathways in a context-dependent manner.

Collectively, current evidence supports a conceptual shift in understanding CA VIII not as an inactive enzyme, but as a functionally active molecular scaffold that regulates key intracellular processes. This paradigm underscores the importance of re-evaluating catalytically inactive proteins within enzyme families, as they may serve essential regulatory roles that are distinct from, yet equally critical as, enzymatic functions.

Future investigations should move beyond descriptive analyses toward mechanistic dissection of CA VIII–mediated signaling networks, particularly its interaction with IP3R1 and associated calcium-dependent pathways. Integrating structural biology with functional and systems-level approaches will be essential to define how CA VIII orchestrates intracellular signaling across different cellular contexts.

Importantly, the study of CA VIII highlights a broader biological principle: the loss of enzymatic activity does not equate to loss of function but can instead drive the evolution of regulatory complexity. In this context, CA VIII serves as a model pseudoenzyme that bridges structural conservation with functional innovation. Elucidating its precise roles may not only advance our understanding of neuronal signaling and disease but also inform the emerging field of pseudoenzyme biology and its therapeutic potential.

## Data Availability

No datasets were generated or analysed during the current study.

## Declarations

### AI Declaration

Artificial Intelligence (AI) tools (ChatGPT, OpenAI) were used solely for language refinement, grammar correction, and improving clarity of expression. No AI tools were used for data generation, analysis, interpretation, or creation of scientific content. All scientific concepts, interpretations, and conclusions are the original work of the author.
